# CLEAR Strategy Inhibited HSV Proliferation Using Viral Vectors Delivered CRISPR-Cas9

**DOI:** 10.3390/pathogens12060814

**Published:** 2023-06-07

**Authors:** Min Ying, Huadong Wang, Tongtan Liu, Zengpeng Han, Kunzhang Lin, Qing Shi, Ning Zheng, Tao Ye, Huinan Gong, Fuqiang Xu

**Affiliations:** 1 State Key Laboratory of Magnetic Resonance and Atomic and Molecular Physics, Key Laboratory of Magnetic Resonance in Biological Systems, Wuhan Center for Magnetic Resonance, Innovation Academy for Precision Measurement Science and Technology, Chinese Academy of Sciences, Wuhan 430071, China; yingmin18@mails.ucas.ac.cn (M.Y.); hanzengpeng17@mails.ucas.ac.cn (Z.H.); zhengning1993@163.com (N.Z.); 2 Key Laboratory of Quality Control Technology for Virus-Based Therapeutics, Guangdong Provincial Medical Products Administration, NMPA Key Laboratory for Research and Evaluation of Viral Vector Technology in Cell and Gene Therapy Medicinal Products, The Brain Cognition and Brain Disease Institute, Shenzhen Institute of Advanced Technology, Chinese Academy of Sciences, Shenzhen 518055, China; ltt2013140@gmail.com (T.L.); kz.lin@siat.ac.cn (K.L.); q.shi@siat.ac.cn (Q.S.); hn.gong1@siat.ac.cn (H.G.); 3 Shenzhen Key Laboratory of Viral Vectors for Biomedicine, Shenzhen-Hong Kong Institute of Brain Science, Shenzhen Institute of Advanced Technology, Chinese Academy of Sciences, Shenzhen 518055, China; 4 University of Chinese Academy of Sciences, Beijing 100049, China; 5 College of Biotechnology, Tianjin University of Science & Technology, Tianjin 300457, China; 6 Chinese Academy of Sciences Key Laboratory of Brain Connectome and Manipulation, Shenzhen Key Laboratory of Translational Research for Brain Diseases, The Brain Cognition and Brain Disease Institute, Shenzhen Institute of Advanced Technology, Chinese Academy of Sciences, Shenzhen-Hong Kong Institute of Brain Science, Shenzhen 518055, China; tao.ye@siat.ac.cn; 7 Jiangsu Key Laboratory of Brain Disease and Bioinformation, College of Life Sciences, Xuzhou Medical University, Xuzhou 221004, China; 8 Center for Excellence in Brain Science and Intelligence Technology, Chinese Academy of Sciences, Shanghai 200031, China

**Keywords:** herpes simplex virus, CRISPR-Cas9, gRNA, lentivirus, *VP16*, *ICP27*, *ICP4*, *gD*

## Abstract

Herpes simplex virus type 1 (HSV-1) is a leading cause of encephalitis and infectious blindness. The commonly used clinical therapeutic drugs are nucleoside analogues such as acyclovir. However, current drugs for HSV cannot eliminate the latent virus or viral reactivation. Therefore, the development of new treatment strategies against latent HSV has become an urgent need. To comprehensively suppress the proliferation of HSV, we designed the CLEAR strategy (coordinated lifecycle elimination against viral replication). *VP16*, *ICP27*, *ICP4*, and *gD*—which are crucial genes that perform significant functions in different stages of the HSV infection lifecycle—were selected as targeting sites based on CRISPR-Cas9 editing system. *In vitro* and *in vivo* investigations revealed that genome editing by *VP16*, *ICP27*, *ICP4* or *gD* single gene targeting could effectively inhibit HSV replication. Moreover, the combined administration method (termed “Cocktail”) showed superior effects compared to single gene editing, which resulted in the greatest decrease in viral proliferation. Lentivirus-delivered CRISPR-Cas9/gRNA editing could effectively block HSV replication. The CLEAR strategy may provide new insights into the potential treatment of refractory HSV-1-associated diseases, particularly when conventional approaches have encountered resistance.

## 1. Introduction

Herpes simplex virus 1 (HSV-1) is the prototype member of the human *Herpesviridae* family. Epidemiological surveys show that HSV-1 is a ubiquitous virus with a global seroprevalence rate of 50–90% in normal individuals [1,2,3], which is the most common causative agent for cold sores, viral encephalitis, and infectious blindness [4,5,6]. HSV-1 usually causes mild and sometimes asymptomatic infections in healthy or immunocompetent individuals but can lead to severe eyelid inflammation and encephalitis caused by severe central nervous system (CNS) infection in neonates or immune-compromised patients [7].

HSV-1 can engage in lytic infection to produce viral progeny and establish a latent infection that can co-exist with the host during the lifetime of the host. HSV-1 is one of the most common causes of encephalitis in children and adults [8,9]. Neurotropic HSV-1 can infect the axon terminals of olfactory neurons, enter CNS via the retrograde axon transport of neurons, and finally reach the olfactory bulb in the brain. HSV-1 can also be reactivated from the neurons in the trigeminal ganglion and be transported anterogradely to the skin or CNS [10]. Herpes simplex encephalitis (HSE) is one of the most serious manifestations of HSV-1 infection, with a mortality rate of 97%. HSV-1 infection in the cornea can cause chronic immune inflammatory disease, namely herpes simplex keratitis (HSK), which will lead to irreversible damage and blindness in the infected patient [11]. Currently, there is no vaccine for preventing HSV infection [3,12]. Both primary viral infection and reactivation after latent infection may cause chronic inflammatory diseases of different degrees [13]. Due to the widespread prevalence of HSV-1, the issue of latent infection is serious. The commonly used clinical therapeutic drugs are nucleoside analogues represented by acyclovir (ACV) [3], which inhibit viral DNA synthesis and are highly effective in blocking viral replication and lytic infection [14]. Although the use of antiviral drugs ACV can reduce the mortality rate of HSE patients, most patients have permanent neurological sequelae after recovery, including symptoms of cognitive, memory and behavioral disorders, and will increase the incidence rate of epilepsy [10,15]. Moreover, they have little effect on viral latency and cannot completely eliminate viral infection. These issues point to a strong need to develop improved and specific strategies for the prevention and treatment of HSV-1 infection, especially for latent viral infection.

HSV-1 is an enveloped double-stranded DNA virus. The genome of HSV-1 is large and complex, consisting of a unique long (UL) region and a unique short (US) region, with reverse repeats on its flanks. HSV genome is about 152 kb, encoding more than 80 different proteins [16]. The diameter of HSV-1 virus particles is about 200 nm. Viral entry initiates with glycoprotein gD binding to one of its receptors: nectin-1, HVEM or modified heparan sulphates [17,18]. Therefore, gD is thought to be the major determinant of HSV tropism and also important in HSV-1 progeny replication and package [19].

Following the viral entry step, viral genomes are released within the nuclei and replicated, and progeny viruses are assembled [20]. During HSV-1 lytic infection, viral genes are expressed sequentially in a cascade fashion, and they are defined by immediate–early (IE), early (E), and late (L) phases [2]. The entire lifecycle of HSV lytic infection is tightly regulated by a collection of viral genes (e.g., *VP16*, *ICP4*, and *ICP27*) [21,22,23], which are essential for HSV-1 replication and lytic infection. VP16 (alpha-gene-transactivating factor, α-TIF) is a viral tegument protein that plays a crucial role in initiating the lytic cycle of HSV. Upon entry into the host cell, VP16 forms a complex with cellular factors Oct-1 and host cell factor 1 (HCF-1), enabling the activation of viral immediate–early (IE) genes. The expression of IE genes is the first step in the HSV lytic cycle, which subsequently leads to the expression of early and late viral genes, viral DNA replication, and the production of progeny viral particles [24]. ICP4 (infected cell polypeptide 4) is an HSV immediate–early protein that functions as a viral transcriptional regulator. ICP4 is essential for the expression of early and late viral genes, as it binds to viral promoters and modulates gene expression. By regulating the transcription of viral genes, ICP4 plays a pivotal role in controlling the progression of the viral lytic cycle and ensuring efficient virus replication [25,26]. ICP27 (infected cell polypeptide 27) is another HSV immediate–early protein that has multiple roles in the viral life cycle, including the transcriptional and post-transcriptional regulation of viral gene expression. ICP27 influences the export of viral mRNA from the nucleus to the cytoplasm and has been shown to interact with cellular splicing factors, potentially affecting viral RNA processing. Additionally, ICP27 may play a role in inhibiting host cell gene expression, thereby promoting viral replication [27].

The genome editing strategy represents a promising approach against viral infection and replication by modifying or destroying the genetic material of viruses [28,29,30,31]. Several studies have used the CRISPR/Cas9 system against HSV-1 productive infection, and gRNAs were constructed to target HSV-1 essential genes, such as *ICP0*, *ICP4*, *ICP27*, *UL8*, *UL29*, *US2*, etc. [32,33,34]. In a recent study, researchers presented evidence demonstrating the effective inhibition of HSV-1 infection *in vitro* using CRISPR-Cas9 and CRISPR-CasX systems, highlighting a promising direction for developing antiviral therapies targeting UL30 [35]. Here, we developed a multi-target genome editing approach against HSV-1 infection, targeting and eliminating HSV-1 genomes via a method named CLEAR (coordinated lifecycle elimination against viral replication). RNA-guided CRISPR/Cas9 genome editing was used to specifically target the DNA sequences of HSV-1 genes *VP16*, *ICP27*, *ICP4*, or *gD*, which express key viral proteins that stimulate HSV-1 gene expression, replication, or packaging [36]. Our findings revealed that lentivirus delivered CRISPR-Cas9/gRNA-mediated targeting *VP16*, *ICP27*, *ICP4*, and *gD* genes reduced HSV-1 infectivity in cell culture models and protected mice against HSV-1 infection. These results support the promise of the CLEAR approach for developing potential anti-HSV-1 therapies that are capable of eliminating latent viral infections.

## 2. Results

### 2.1. CLEAR Strategy Design and Lentivirus Construction

**Figure 1:**
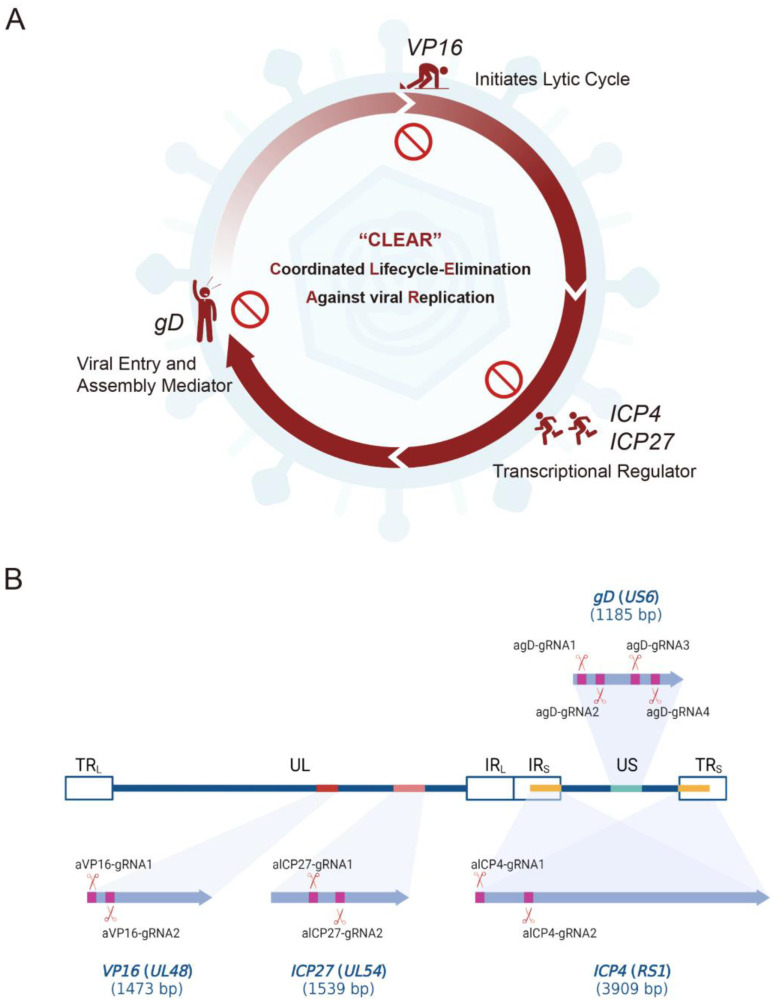
The schematic diagram of the CLEAR (coordinated lifecycle elimination against viral replication) strategy and the designed gRNAs targeting essential HSV1 genes. (**A**) Schematic representation of the CLEAR strategy. *VP16*, *ICP27*, *ICP4*, and *gD* play different essential roles in the different stages of the HSV proliferation cycle. (**B**) The schematics of 10 designed gRNAs and their targeting and cleavage sites. The process involves utilizing lentiviral vectors to deliver SpCas9 and viral gene-targeting gRNAs, ultimately aiming to eliminate key viral genes and inhibit HSV replication.

To block HSV viral proliferation cycle, viral essential genes *VP16*, *ICP27*, *ICP4*, and *gD* were selected as the potential targets for editing by the CLEAR strategy based on CRISPR/Cas9 (Figure 1A). We designed sgRNAs targeting four essential viral genes and developed guide RNAs to combine with Cas9 in order to test their ability in suppressing HSV-1 replication (Figure 1B).

**Figure 2:**
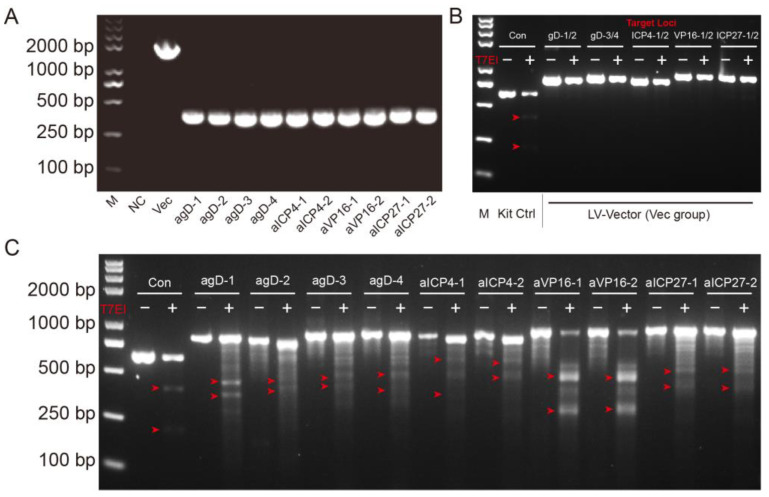
Gene verification and editing activity analysis of constructed LV-Cas9-gRNA plasmids. (**A**) PCR confirmation of the correct gRNA cassette integration into the lentiCRISPR-v2 vector. Lane 1, molecular size marker (M); Lane 2, negative control; Lane 3, 1885 nucleotide fragments consistent with the parental vector amplified product (mesoplasma florum L1 gene) size; Lanes 4–13, the right amplified products of constructed lentiviral plasmids consistent with correct the insertion of the gRNAs cassette. (**B**,**C**) Mutation detection in targeted cells by T7E1 assay. BHK-21 cells were transfected with 2 μg of LV-Vec or LV-Cas9-gRNA plasmids and subsequently infected with H129-EGFP at MOI = 0.01 24 h later. Cells were harvested 48 h post infection, and lysates were used to amplify target loci. Amplified products were denatured/annealed and digested with T7 Endonuclease I according to the recommended protocol. The resulting fragments were consistent with the expectation. The corresponding fragment sizes, as marked by the red arrows, were as follows: Con, 514 bp = 335 bp + 179 bp; *agD-1*, 803 bp = 436 bp + 367 bp; *agD-2*, 803 bp = 410 bp + 393 bp; *agD-3*, 837 bp = 418 bp + 419 bp; *agD-4*, 837 bp = 467 bp + 370 bp; *aICP4-1*, 910 bp = 545 bp + 365 bp; *aICP4-2*, 910 bp = 521 bp + 389 bp; *aVP16-1*, 900 bp = 517 bp + 383 bp; *aVP16-2*, 900 bp = 497 bp + 403 bp; *aICP27-1*, 897 bp = 521 bp + 376 bp; *aICP27-2*, 897 bp = 481 bp + 416 bp.

sgRNAs were designed based on the target site sequence of selected genes and were screened by the CRISPR DESIGN system (http://crispr.mit.edu/ (accessed on 15 October 2021)). sgRNA oligonucleotide sequences are shown in Table 1. A total of 10 gRNAs were designed: VP16 targeting (two gRNAs), ICP27 targeting (two gRNAs), ICP4 targeting (two gRNAs), and gD targeting (four gRNAs) (Figure 1B, Table 1). The corresponding 10 core plasmids of lentiviral vectors carrying SpCas9 and gRNAs were constructed and verified (Figure 2A). Then, the preferred lentiviruses were packaged and prepared using standard procedures for the gene delivery of the CRISPR editing system.

The T7 Endonuclease Mutation Detection assay was used to evaluate the editing activity of selected gRNAs at the genome level. BHK-21 cells were transfected with lentiviral vector or Cas9-gRNA plasmids and infected with HSV H129-EGFP 24 h later. Genomic DNA was isolated from BHK-21 cells, and targeted loci were amplified, denatured/annealed, and digested with T7 Endonuclease I (T7E1), a structure-specific enzyme that will recognize mismatches larger than one base. No digested bands were observed in the control (Vec) group (Figure 2B). The resulting digested fragments of Cas9-gRNA editing were observed and consistent with the design expectation (Figure 2C). The results demonstrated that 10 designed gRNAs combined with SpCas9 could efficiently and accurately target and cut target genes.

### 2.2. In Vitro Experiments to Verify the Effect of Different gRNA Editing to Suppress HSV Replication

**Figure 3:**
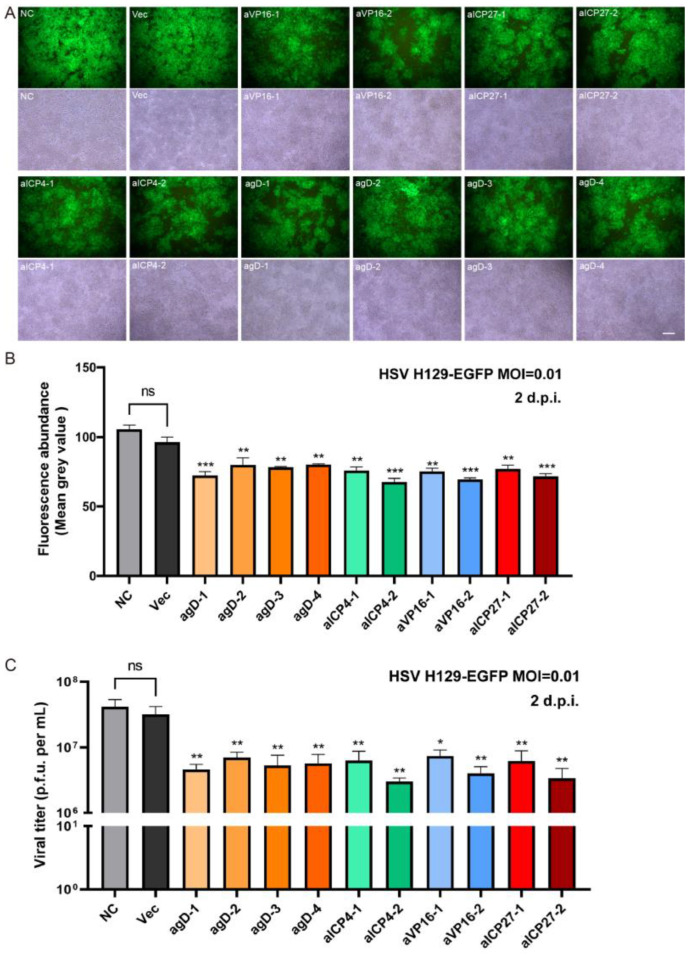
The inhibitory effect of CRISPR single gene editing on HSV replication *in vitro*. (**A**) Cas9-gRNA editing inhibited HSV replication in BHK-21 cells. Different Cas9/gRNA plasmids were transfected into BHK-21 cells, and cells were infected with HSV H129-EGFP 24 h later. Cell fluorescence images were collected 48 h after HSV infection (MOI = 0.01). The bright field images show the total number of cells, complete confluence, and inter-group consistency of the results. Scale bar = 200 μm; (**B**) the fluorescence abundance (average gray value) of EGFP expression at 48 h post-infection. (**C**) The viral titers of HSV were determined. Statistical values were presented as mean ± SEM. Significant differences were expressed by the *p* value. ns, no significant difference; * *p* < 0.05, ** *p* < 0.01, *** *p* < 0.001 (sgRNA versus Vec).

In order to validate the capacity of distinct gRNAs to suppress HSV replication, engineered gRNA plasmids were independently transfected into BHK-21 cells. Subsequently, these cells were infected with HSV H129-EGFP at an MOI of 0.01 following a 24 h period. The inhibitory effects of various gRNAs were evaluated by monitoring the expression intensity of the green fluorescent gene, which served as a reporter. In comparison to the control groups (NC or Vec), all groups transfected with sgRNA plasmids exhibited a reduction in fluorescence intensity (Figure 3A,B).

Furthermore, the viral titer of HSV serves as an additional indicator of gRNA efficacy. A significant reduction in HSV titers was observed in gRNA-transfected groups compared to the control group. The average titer of the VEC control group is 3.19 × 10^7^ PFU/mL, and the titers of the experimental groups have generally decreased. The greatest effect was observed in the aICP4-2 group with a decrease in titer of 3.03 × 10^6^ PFU/mL, which is 10 times lower than the control. The average titer of the VP16-1 group decreased the least, with a decrease of about 4.3 times, to a titer of 7.41 × 10^6^ PFU/mL (Figure 3C). Among the groups, agD-1, aICP4-2, aICP27-2, and aVP16-2 demonstrated the most potent inhibitory effects on HSV proliferation, prompting their selection for subsequent experiments and lentiviral packaging. Additionally, the inhibitory effects of the designed gRNAs targeting HSV replication were further corroborated using 293T cells (a human renal epithelial cell line) (Figure S1).

### 2.3. Enhanced Inhibitory Effect of the CLEAR Strategy on HSV Compared to Individual gRNAs Exhibiting a Dose–Response Relationship

**Figure 4:**
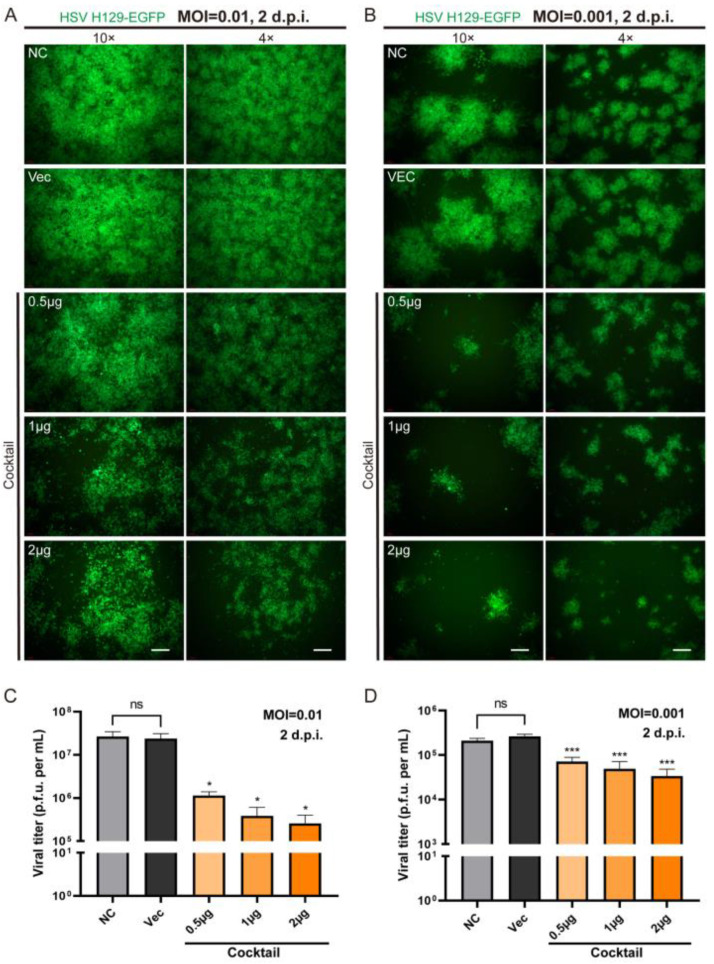
The efficacy of the CLEAR strategy in inhibiting HSV replication *in vitro*. (**A**) Fluorescence images of cells 48 h post cocktail transfection following HSV infection (MOI = 0.01), with both 4× and 10× magnification images. (**B**) Fluorescence images of cells 48 h post-cocktail transfection following HSV infection (MOI = 0.001), with both 4× and 10× magnification images. Images at 4×; scale bar = 500 μm. Images at 10×; scale bar = 200 μm. (**C**) The viral titers of HSV infection (MOI = 0.01) 48 h after cocktail transfection; cocktail versus Vec. ns, no significant difference; * *p* < 0.05. (**D**) The viral titers of HSV infection (MOI = 0.001) 48 h after cocktail transfection; cocktail versus Vec. ns, no significant difference; *** *p* < 0.001. The statistically significant reduction in viral titers after cocktail transfection further supported the efficacy of the CLEAR strategy in inhibiting HSV replication *in vitro*.

Given the rapid proliferation efficiency of HSV despite the use of single gRNA editing, the CLEAR (coordinated lifecycle elimination against viral replication) strategy was proposed, involving the co-transfection of four gRNA plasmids (agD-1, aICP4-2, aICP27-2, and aVP16-2), referred to as a cocktail. Three dose gradients of 0.5 µg, 1 µg, and 2 µg were established, and HSV H129-EGFP was infected 24 h post transfection at MOIs of 0.01 and 0.001. The fluorescence images showed a clear reduction in fluorescence intensity in the cocktail group compared to the Vec control group, indicating decreased HSV replication. Whether assessed by HSV titers or reporter gene expression levels, the cocktail approach effectively suppressed HSV proliferation under both MOI conditions of 0.01 (Figure 4A,C) and 0.001 (Figure 4B,D).

Moreover, the HSV titer of the cocktail (0.5 µg) group decreased approximately 21-fold (2.37 × 10^7^ PFU/mL to 1.13 × 10^6^ PFU/mL) compared to the Vec control (Figure 4C, 0.5 µg), which was double the inhibitory effect of the same dose of a single gRNA, which decreased about 10-fold (3.19 × 10^7^ PFU/mL to 3.03 × 10^6^ PFU/mL) (Figure 3B, aICP4-2). These findings suggested that the CLEAR strategy was more effective than individual gRNAs in inhibiting HSV replication *in vitro*. Furthermore, the cocktail approach demonstrated significant statistical differences between various dosage groups, indicating the presence of a dose–response relationship for the cocktail approach in this experimental series (Figure 4C,D).

### 2.4. The CLEAR Strategy Exhibits Preventive and Therapeutic Potential

**Figure 5:**
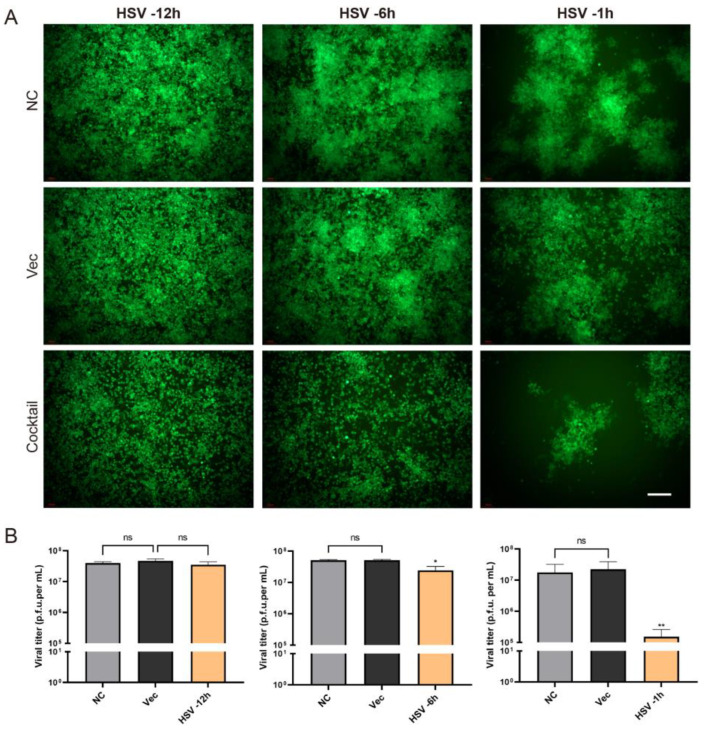
The protection effect of the CLEAR strategy after HSV1 pre-infection. (**A**) The cell fluorescence images of cocktail transfection groups at 1 h, 6 h, and 12 h after HSV infection. Scale bar = 200 μm. (**B**) The viral titers of gRNA cocktail editing groups at 1 h, 6 h, and 12 h after HSV infection (MOI = 0.01). ns, no significant difference; * *p* < 0.05; ** *p* < 0.01.

To emulate the treatment of HSV-infected patients, cells were initially infected with HSV (MOI = 0.01), followed by co-transfection with the four gRNA plasmids (cocktail) at 1 h, 6 h, and 12 h post HSV infection, respectively. The inhibitory effects of the cocktail were evaluated by the HSV titer and reporter gene expression levels. The cocktail administration at 1 h.p.i. demonstrated the most potent inhibitory effect compared to 6 h.p.i. and 12 h.p.i. timepoints (Figure 5A,B). Notably, the administration of the cocktail 12 h after HSV infection did not produce any discernible inhibitory effect. However, in the experimental group given 1 h.p.i, the proliferation efficiency of HSV was inhibited 100-fold (Figure 5B). These findings indicated that the timing of cocktail administration is one of the main factors for its effectiveness in suppressing HSV replication. The significant inhibitory effect observed at the 1 h.p.i. timepoint suggests that the cocktail editing approach holds potential for therapeutic applications, providing protection against HSV infection when administered promptly. Moreover, the reduced inhibitory effects at the 6 h.p.i. timepoints imply that the cocktail editing approach may still offer some therapeutic benefits when administered during the early stages of infection.

### 2.5. CLEAR Strategy Could Effectively Inhibit HSV Replication and Transmission In Vivo

To evaluate the inhibitory effect of Cas9/gRNA editing on HSV *in vivo*, we designed experiments in which mice were intracerebrally inoculated with HSV1 H129-EGFP after the pre-administration of lentiviral vectors. The goal was to verify the inhibitory effect of lentiviral vectors carrying gene-editing elements on HSV replication and transmission in the brains of C57BL/6 mice.

**Figure 6:**
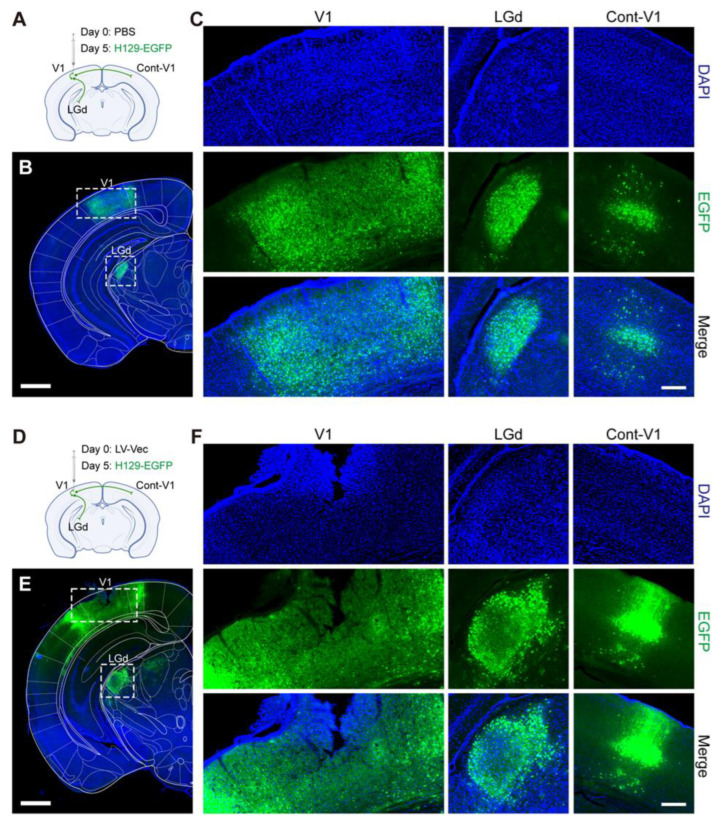
The control mice received either PBS or LV-Vec showed strong HSV1 replication and spread after viral challenge. (**A**) Diagram illustrating circuit connections and the infusion of PBS and H129-EGFP. The dots on the green lines represent the cell body of the neuron, and the end of the green lines represent the axon terminals. V1, primary visual area; LGd, dorsal part of the lateral geniculate complex; Cont-V1, contra-lateral V1. PBS (500 nL) and H129-EGFP (5 × 10^2^ PFU) were injected 5 days apart (n = 3). (**B**) The dissemination of HSV1 H129-EGFP viral infection in the PBS control group. Scale bar = 1 mm. (**C**) The fluorescence distribution of EGFP at the injection site of V1 and downstream areas of LGd and contra-lateral V1. Scale bar = 200 μm. (**D**) Diagram illustrating circuit connections and the viral infusion of LV-Vec and H129-EGFP. LV vector control (LV-Vec, 5 × 10^4^ TU) and H129-EGFP (5 × 10^2^ PFU) were injected 5 days apart. (**E**) The dissemination of HSV1 H129-EGFP viral infection in the LV-Vec control group. Scale bar = 1 mm. (**F**) The fluorescence distribution of EGFP at the injection site of V1 and downstream areas of LGd and contra-lateral V1. Scale bar = 200 μm.

PBS and LV-Vec were administrated as controls, respectively (Figure 6A,D), and pre-injected in the primary visual area (V1); then, H129-EGFP was injected 5 days later. EGFP-positive cells could be observed both at the injection site (V1) and the downstream output areas such as the dorsal part of the lateral geniculate complex (LGd) and contra-lateral V1 (Cont-V1) 5 days after the reporter’s gene expression (Figure 6B,C,E,F and Figure S2).

**Figure 7:**
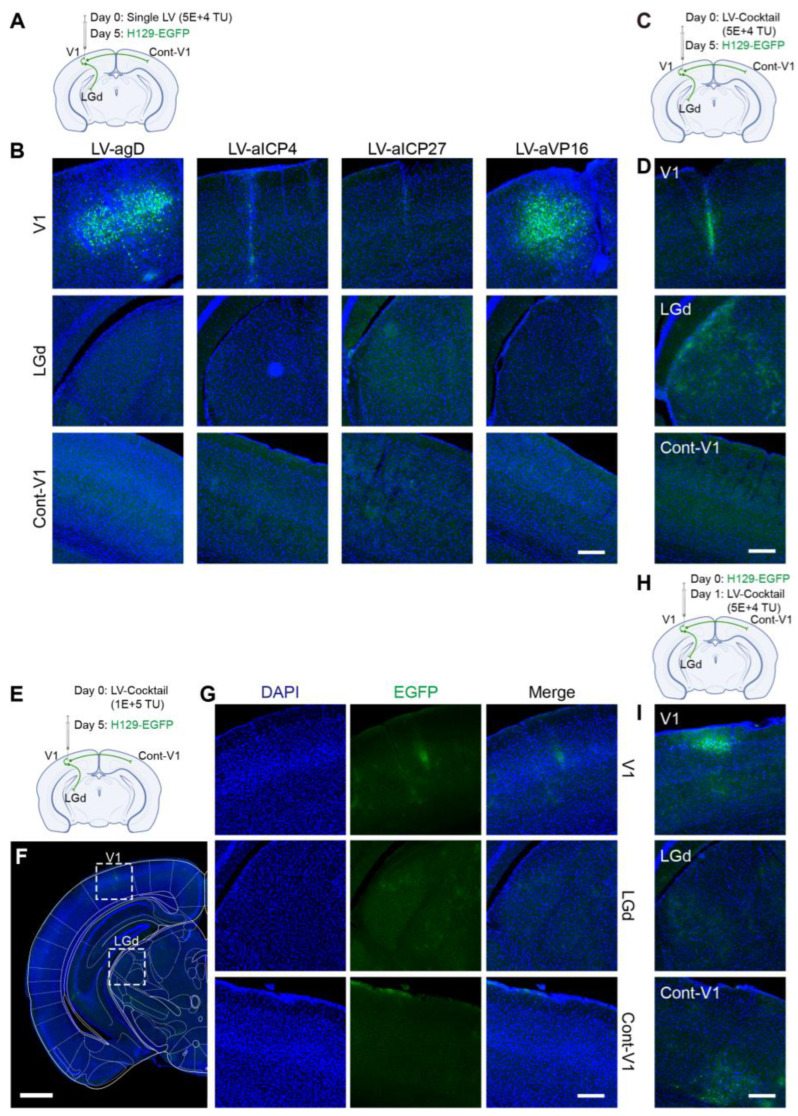
Lentiviral vectors delivering gene-editing elements effectively cleared HSV1 H129 *in vivo*. We designed four experimental groups: (**A**,**B**) four individual LV treatments, (**C**,**D**) a mixed treatment group (LV-Cocktail) with equal amounts of each single LV, (**E**–**G**) a double-concentration mixed treatment group (LV-Cocktail), and (**H**,**I**) a group inoculated with HSV first and then injected with LV. (**A**) Diagram illustrating circuit connections and the viral infusion of HSV1 H129-EGFP and the single LV vector. Single LV (5 × 10^4^ TU) and H129-EGFP (5 × 10^2^ PFU) were injected 5 days apart (n = 3). (**B**) The fluorescence distribution of EGFP at the injection site of V1 and downstream areas of LGd and contra-lateral V1 via the single administrated LV. Scale bar = 200 μm. (**C**) Diagram illustrating circuit connections and the viral infusion of HSV1 H129-EGFP and low doses of the LV-Cocktail. LV-Cocktail (5 × 10^4^ TU) and H129-EGFP (5 × 10^2^ PFU) were injected 5 days apart. (**D**) The fluorescence distribution of EGFP at the injection site of V1 and downstream areas of LGd and contra-lateral V1. Scale bar = 200 μm. (**E**) Diagram illustrating circuit connections and the viral infusion of HSV1 H129-EGFP and high-dose LV-Cocktail. LV-Cocktail (1 × 10^5^ TU) and H129-EGFP (5 × 10^2^ PFU) were injected 5 days apart. (**F**) The dissemination of HSV1 H129-EGFP after clearance by high-dose LV vectors 5 days later; scale bar = 1 mm. (**G**) The fluorescence distribution of EGFP at the injection site of V1 and downstream areas of LGd and contra-lateral V1 via high dose of administrated LV. Scale bar = 200 μm. (**H**) Diagram illustrating circuit connections and the viral infusion of HSV1 H129-EGFP and the LV-Cocktail vector (LV-Cocktail administration after first infection with HSV). H129-EGFP (5 × 10^2^ PFU) and LV (5 × 10^4^ TU) were injected 1 day apart. (**I**) The fluorescence distribution of EGFP at the injection site of V1 and downstream areas of LGd and contra-lateral V1. Scale bar = 200 μm.

Based on the results of the *in vitro* experiments, lentiviruses LV-agD-1, LV-aICP4-2, LV-aICP27-2, and LV-aVP16-2 were selected and packaged. Four LV vectors were administered individually (Figure 7A) and pre-injected in the V1 brain region, and H129-EGFP was injected 5 days later compared with the control groups. After the administration of LV vectors, EGFP-positive cells were significantly reduced ^in situ^; in particular, the LV-aICP4 and LV-aICP27 groups had few EGFP-positive neurons at the injection site, and there was almost no fluorescence signal in the downstream brain areas (LGd and Cont-V1) (Figure 7B and Figure S3).

Furthermore, we used the LV-Cocktail at low (Figure 7C,D) and high (Figure 7E–G) doses to verify the clearance effect on HSV; except for the trace fluorescence expression at the local area, the fluorescence signal was difficult to observe in the rest of the brain regions (Figure 7D,G and Figure S4A,B).

In addition, we also tested the inhibitory effect of the LV-Cocktail when HSV infection was done first (Figure 7H,I). The significant inhibition of HSV proliferation and transmission could also be observed by using LV vectors carrying gene-editing elements on the day after the H129 infection (Figure 7I and Figure S4C). The results demonstrated that lentivirus delivered Cas9/gRNA-mediated targeting *VP16*, *ICP27*, *ICP4*, and *gD* genes apparently inhibited HSV replication and protected mice against HSV challenge. Moreover, the CLEAR strategy effectively inhibited HSV virus replication and transmission.

## 3. Discussion

Herpes cold sores caused by HSV-1 infection will occur repeatedly, which can be attributed to the lifelong persistent infection of the virus in the host [37,38]. This strategy is crucial for the survival of viruses because the entire life process of the host provides a repository for regular viral reactivation. When the host’s immunity declines, the virus recurs in the patient via reactivation. Relevant studies indicate that 70% of HSE cases in adults are attributable to previous HSV-1 infection and virus reactivation [4].

HSV-1 therapy consists of nucleotide analogs such as acyclovir (ACV) and valacyclovir. Since the 1980s, ACV and its derivatives have become the first choice for the prevention and treatment of herpes virus infection [3]. The continuous emergence of drug-resistant virus cases also poses higher requirements for antiviral treatment. Consequently, the issue of drug resistance warrants significant attention, underscoring the urgent need for research and development of novel therapeutic strategies. Increasing evidence shows that CRISPR technology can be used to effectively inhibit virus replication, including HSV, SARS-CoV-2 [39], HBV [40], HIV [41], etc. Due to the large size of the Cas9 gene, a viral gene delivery vector with a large load capacity is required. Currently, AAV and lentivirus are the most commonly used viral vector candidates. Considering the limited gene-carrying capacity of AAV vectors, utilizing lentiviral vectors to deliver CRISPR elements may present a viable approach for herpes simplex keratitis (HSK) treatment. This prospective strategy could be applied through corneal therapy. Beyond merely eliminating HSV at the local corneal level, the lentivirus vector can retrogradely be transported along the axon and infect trigeminal ganglion neurons, and subsequently disrupting HSV reservoirs within the trigeminal ganglion [32].

In this study, we assessed the ability of CRISPR/Cas9 editing to eliminate HSV-1 replication by targeting specific DNA sequences essential to viral protein expression during the early and late phases of viral infection/reactivation. We designed specific gRNAs targeting the essential genes of HSV—*VP16* (2 gRNAs), *ICP27* (2 gRNAs), *ICP4* (2 gRNAs), and *gD* (4 gRNAs)—and constructed 10 core plasmids of lentiviral vectors carrying SpCas9 and the corresponding gRNA (Figure 1); then, we packaged and prepared lentiviruses for the gene delivery of the CRISPR editing system. First of all, we screened the inhibitory effect of the HSV replication of 10 gRNA-mediated *in vitro* editing instances. Two days after infection with the H129-EGFP virus, the viruses in the control groups (NC and Vec) proliferated rapidly, and almost all cells expressed green fluorescent proteins, while the fluorescence ratio of single gRNA groups decreased to varying degrees (Figure 3A). By determining the virus titers of the cell supernatant, all viral loads of the Cas9/gRNA groups were significantly lower than that of the control groups. Four types of gRNA—agD-1, aICP4-2, aVP16-2, and aICP27-2—were observed to have the most obvious inhibitory effect (Figure 3B), thus serving as candidates for producing lentivirus vectors. At present, lentiviral vectors are widely used in CAR-T cell therapy to transduce patient-derived T cells *in vitro*. For gene therapy, the viral genome of lentivirus will be integrated into the host genome, which is one of the main safety risks. However, the lentiviral vector also has certain advantages. As a pseudovirus vector, it is less toxic. The lentivirus itself does not proliferate, and the risk is controllable. Moreover, Cai et al. reported that HSV elimination in the TG reservoirs could be achieved by lentivirus-mediated CRISPR editing via the lentivirus retrogradely transport from the cornea to the trigeminal ganglion [32]. Of course, it is safer to choose the recombinant adeno-associated virus (rAAV) virus vector. However, the disadvantage is that the carrying capacity of rAAV is too small for delivering CRISPR system.

In order to more effectively block the life cycle of HSV replication by multi-point genome editing, we tested whether the combination of Cas9/gRNAs (CLEAR strategy) simultaneously targeting four genes could achieve improved inhibition effects against HSV replication. We applied different concentrations (0.5 μg, 1 μg, and 2 μg) of cocktail gRNA plasmids to transfect cells, and then the HSV1 H129-EGFP virus infected the transfected cells at MOI = 0.01 and MOI = 0.001 (Figure 4). It was found that the cocktail groups showed significant inhibition on HSV replication, even at low doses. With the dose increase in Cas9/gRNA plasmids, the inhibition of viral proliferation was stronger (Figure 4A,C). These results indicated that the combined utilization strategy of Cas9/gRNA targeting the four genes could effectively inhibit HSV replication and proliferation.

As described above, we tested the efficiency of the CRISPR editing system in preventing HSV infection. What was the therapeutic effect of the editing system? In order to solve this problem, we carried out an experiment in which the cells were first infected with HSV H129-EGFP and then treated the cells with CRISPR editing. H129-EGFP viruses were challenged 12 h, 6 h, and 1 h in advance and then transfected with Cas9/gRNA cocktail plasmids. It was found that the Cas9/gRNA intervention had a certain inhibition effect but was not obvious in the robust proliferation stage of HSV acute infection (−12 h) (Figure 5). If CRISPR intervened earlier after HSV infection, the CRISPR editing treatment in the −6 h group and −1 h group could significantly reduce HSV replication; the earlier it intervenes, the better.

Subsequently, *in vivo* animal experiments with the produced lentiviruses were carried out. It was found that the Cas9/gRNA (Cocktail) strategy could effectively inhibit virus proliferation and transmission. In the control groups without any intervention (NC, Figure 6A,B) and the LV-Vec control group (Vec, Figure 6D,E), HSV had effectively proliferated for 5 days after H129-EGFP was injected into the V1 brain region of mice, and it spread to LGd and other brain regions along the neural circuit. The infected neurons were labeled by a green fluorescent protein, and the LV-Vec treatment had no inhibitory effect. When the designed lentivirus carrying Cas9/gRNA was administrated alone (Figure 7A,B), the inhibitory effect on HSV replication (few fluorescent-labeling cells at the injection site) and virus transmission (no fluorescent-labeling cells in the LGd region) were observed, which was better in the LV-aICP4 and LV-aICP27 groups compared with the LV-agD and LV-aVP16 groups. The treatment effect of the Cas9/gRNA combination (Cocktail) group was relatively the best. There were no or only a small number of fluorescent-labeling cells at injection site V1 (Figure 7D,G). Furthermore, we also performed the therapeutic effect experiment. H129-EGFP was first injected in the V1 brain region of mice, and LV-cocktail was administrated 1-day post-infection; it was found that the cocktail treatment could effectively inhibit HSV replication (Figure 7H,I), and the effect was better than that of a single lentivirus administration.

In this study, we developed an antiviral strategy comprising coordinated lifecycle elimination against viral replication (CLEAR). This strategy is different from targeting a key viral gene to inhibit viral genome replication. Instead, it starts with analyzing the characteristics of the viral life cycle and selects a multi-pronged approach to block various life cycle stages of the virus. In this way, it can effectively avoid the disability of single-point genome editing once the virus genome mutates. Multi-point genome editing can simultaneously target multiple key gene targets, thereby effectively inhibiting viral genome replication and producing a synergistic antiviral effect. This method is effective for both proliferative viruses and latent infected viruses, as it involves editing and cutting viral genomic materials. As mentioned above, the CLEAR strategy using CRISPR targeting the key HSV genes—*VP16*, *ICP27*, *ICP4*, and *gD*—could effectively inhibit HSV replication and proliferation, and the combination of Cas9/gRNAs strategy was more efficient. In order to effectively target latent HSV 1-containing neurons, the administration strategies include a local administration method or LV administration at the axonal terminals of neurons. Lentivirus delivers the CRISPR system to enter the cell body of HSV1 latently infected neurons in order to carry out the gene-cutting function in the nucleus, which can effectively prevent viral genome replication in latent cells and achieve the fundamental elimination of the latent virus. In addition, it is worth noting that there was no significant difference in the effect of agD, aICP4, aVP16, and aICP27 editing on inhibiting HSV replication *in vitro*. However, in *in vivo* experiments, the effect of the targeted editing of ICP4 or ICP27 alone was better than that of editing gD and VP16 (Figure 7B). The effectiveness of editing ICP4 or ICP27 in inhibiting HSV replication was consistent with previous reports [14,31,32,34].

In the present study, we assessed the efficiency of Cas9/gRNA genome editing to inhibit HSV replication at the preventive and therapeutic levels. In order to explore the accessibility of gRNAs to their target sequences and determine the time window for CRISPR gene editing, we designed the experiments involving the “prevention strategy” and “treatment strategy” and compared their effects. The results showed that the CRISPR editing system could inhibit HSV replication more effectively by expressing gRNAs in the cells before infection. In the productive life cycle of HSV viruses, when the viral genome is in a naked DNA state, it can certainly be easily recognized, captured, and cut by the Cas9/gRNA complex. When the viral genome is in the form of chromatin, it will increase the difficulty of editing to a certain extent. On one level, the cocktail of gRNA editing and cutting (multiple targeting effects of CLEAR strategy) could provide enhanced efficacy in preventing viral genome replication. Moreover, multi-target blockades could effectively prevent the occurrence of ineffective single-target editing interventions during viral gene mutations. The determination and optimization of the CRISPR gene editing time window need to be further studied.

In conclusion, the study demonstrated that the CRISPR gene editing strategy had a good inhibitory effect in the experiments of both “prevention strategy” and “treatment strategy”, especially *in vivo* experiments. Compared with the control groups, Cas9/gRNA treatments, whether administered alone or combined, significantly inhibited HSV proliferation and spread. The demonstrated dose–response relationship and the applicability of the CLEAR strategy across different MOIs further highlight its potential for developing novel anti-HSV therapies.

## 4. Materials and Methods

### 4.1. Animals

All husbandry and experimental procedures in this study were approved by the Animal Care and Use Committees at the Shenzhen Institute of Advanced Technology or Innovation Academy for Precision Measurement Science and Technology, Chinese Academy of Sciences (approval No. SIAT-IACUC-200902-NS-WHD-A1438). Adult male C57BL/6 mice were purchased from Hunan SJA Laboratory Animal Company. Adult (8–10 weeks old) C57BL/6 mice (male, n = 27) were used for *in vivo* experiments. Mice were randomly assigned to groups of predetermined sample size. No mice were excluded from these analyses. All animals were housed in a dedicated housing room with a 12/12 h light/dark cycle, and food and water were available ad libitum. All the experiments with viruses were performed in biosafety level 2 (BSL-2) laboratory and animal facilities.

### 4.2. Cell and Virus

The wildtype HSV-1 H129 clinical strain was generously provided by Professor Lynn Enquist (Princeton University, Princeton, NJ, USA). Viral stocks were grown on BHK-21 cells (American Type Culture Collection) maintained in Dulbecco’s minimum essential media (DMEM) with 10% fetal bovine serum (FBS). Standard plaque assays were performed by serially diluting virus in DMEM supplemented with 2% FBS (2% DMEM) and overlaying infected cells with medium containing 2% FBS, antibiotics, and 1% agarose. Plaques were identified using neutral red staining and/or fluorescence microscopy where appropriate. Aliquots of viral stocks were stored frozen at −80 °C.

HSV1 H129-EGFP recombinant virus was constructed and maintained in our lab, in which an EGFP expression cassette (containing hUbC promoter, EGFP gene, WPRE and bGH ploy (A) signal) was inserted into the intergenic region between the UL37 and UL38 genes of H129 viral genome. The recombinant H129-EGFP viruses were mass-produced by infecting BHK-21 cells grown in T75 tissue culture flasks. After infected cells showed a prominent cytopathic effect (~2 days), medium containing the viruses was collected, centrifuged to remove cell debris (7000× *g* for 10 min), the supernatant passed through a 0.22 μm filter, and finally centrifuged at 50,000× *g*/3 h using Beckman Avanti J-26SXP Ultracentrifuge. Dissolved viruses were aliquoted into 3 μL and stored at −80 °C. The titer of viral stocks was determined using standard plaque assay and titers were expressed as plaque-forming units (PFU) per milliliter. A fresh aliquot of stock virus was thawed and used for each experiment. The titers of HSV1 H129-EGFP stocks used in these studies were ~2 × 10^9^ PFU/mL.

### 4.3. gRNA and Lentiviral Vector Construction

Lentiviral delivery of both SpCas9 and sgRNA (lenti-CRISPR) for targeted gene editing was constructed. sgRNA was designed based on the target site sequence of selected genes and screened by CRISPR DESIGN system (http://crispr.mit.edu/ (accessed on 15 October 2021)). sgRNA oligonucleotides sequences were as Table 1. The empty vector lentiCRISPR v2 vector (addgene, #52961) was digested using BsmBI, and the annealed oligos were cloned into the single guide RNA scaffold. Then, lentiCRISPR-sgRNA virus was produced in HEK293T cells with the packaging plasmids as previously described methods [42]. In brief, the above lentivirus constructs were co-transfected with three lentivirus packaging vectors (pGAG/POL, pREV and pMD2.G) into 293T cells, and the targeting viruses were obtained by collecting supernatants after 2 to 3 days. The titers of produced five lentiCRISPR-sgRNA (LV-vec, LV-agD-1, LV-aVP16-2, LV-aICP4-2, LV-aICP27-2) viral stocks were all ~1 × 10^8^ PFU/mL.

### 4.4. Infection and Transduction of Cells

BHK-21 cells and 293T cells were seeded in 6-well plates at density of 1 × 10^6^ cells per dish 24 h before 2 μg lentiCRISPR-sgRNA transfection. Six hours after transfection, the medium was refreshed with 2 mL DMEM supplemented with 2% FBS (Gibco, Thermo Fisher, Sydney, Australia) and 1% penicillin/streptomycin (BasalMedia, Shanghai, China), then BHK-21 cells were infected with H129-EGFP at MOI = 0.01 after 18 h. The supernatants were harvested 48 h after infection.

Next, we selected agD-1, aICP4-2, aICP27-2 and aVP16-2 sgRNA in combination of 0.5 μg, 1 μg and 2 μg to transfection BHK-21 cells. Six hours after transfection, the medium was refreshed with 2% DMEM, then BHK-21 cells were infected with various MOI (0.01 or 0.001) of H129-EGFP after 18 h. The supernatants were harvested 24 h and 48 h after infection.

For treatment strategy experiment, BHK-21 cells were infected with MOI at 0.01 of H129-EGFP. After 1 h, 6 h and 12 h infection, BHK-21 cells were transfected with four sgRNAs (agD-1, aICP4-2, aICP27-2 and aVP16-2) in combination of 1 μg. Six hours after transfection, the medium was refreshed with 2% DMEM, then the supernatants were harvested 42 h.

### 4.5. T7 Endonuclease Mutation Detection Assay

The EnGen Mutation Detection Kit (New England Biolabs, Ipswich, MA, USA, M0302) was used for detection of on-target genome editing events. BHK-21 cells were transfected with 2 μg Cas9-gRNA plasmids. Then, 24 h later, the cells were infected with H129-EGFP at MOI = 0.01. The cells were harvested 48 h after infection and lysed with 100 μL QuickExtract™ (Cambio/Epicentre, QE09050) per well and mix by pipetting. A 1 μL volume of lysates was used for the amplification of the target site. Put on PCR plate with 2 μL NEB Buffer and add ddH_2_O to a final volume of 19 μL. Run Cross-Hybridisation PCR program. After products had cross-hybridized add 1 μL T7 endonuclease I in each sample. Incubate at 37 °C for 15 min. Stop T7 endonuclease reaction straight after the end of the incubation period by adding 2 μL 0.25 M EDTA. Add loading buffer to all samples and load them on the 1.5% agarose gel. Run at 100 V and check the gel regularly under the UV, taking images at different time points.

### 4.6. Viral Titer Assay

H129-EGFP titer was performed in triplicate for each biological sample. BHK-21 (2 × 10^5^) cell was seeded in 6-well plate, then infected BHK-21 with various dilution of virus supernatant on the following day. After 1 h of infection, 0.5% agarose (Biowest agarose, Baygene, Shanghai, China) in DMEM supplemented with 2% FBS (Gibco, Thermo Fisher, Australia) and 1% penicillin/streptomycin (BasalMedia, Shanghai, China, S110JV) was added. The plaques were counted after 48 h incubation in 35 °C, 5% CO_2_ Cell Culture Chamber.

### 4.7. Stereotactic Surgery

All procedures on animals were performed in Biosafety level 2 (BSL-2) animal facilities [43]. Animals were anesthetized with pentobarbital sodium by intraperitoneal injection (80 mg/kg, i.p.), and placed in a stereotaxic apparatus (RWD, Shenzhen, China, 68030). During surgery and virus injection, all animals were kept anesthetized with isoflurane (1–1.5%). The skull above the targeted areas was thinned with a dental drill and removed carefully. Injections were conducted with a syringe pump (Stoelting, Wood Dale, IL, USA, 53311) connected to a glass micropipette with a tip diameter of 10–15 mm. The glass micropipette was held for an extra 10 min after the completion of the injection and then slowly retreated. After the surgery, the incisions were stitched and lincomycin hydrochloride and lidocaine hydrochloride gel was applied to prevent inflammation and alleviate pain for the animals. 5 × 10^4^ TU lentiCRISPR-gRNA viruses and 500 PFU H129-EGFP viruses was injected into the adult male C57BL/6 mice (n = 3) at 0 d.p.i. and 5 d.p.i. respectively, with the following coordinates: V1 (AP, −2.80 mm; ML, −2.40 mm; and DV, −0.90 mm).

### 4.8. Slice Preparation and Confocal Imaging

Five days post injection with HSV H129-EGFP, the mice were anesthetized with pentobarbital sodium (100 mg/kg body weight, i.p.), and perfused transcardially with PBS (5 min), followed by ice-cold 4% paraformaldehyde (PFA, 158127 MSDS, sigma) dissolved in PBS (5 min). The brain tissues were carefully removed and post-fixed in PBS containing 4% PFA at 4 °C overnight, and then equilibrated in PBS containing 25% sucrose at 4 °C for 72 h. The 40-μm-thick coronal slices of the whole brain were obtained using the cryostat microtome and stored at −20 °C.

For all samples, every sixth section of the brain slices were selected, stained with DAPI, washed with PBS, mounted with 70% glycerol (in PBS) and sealed with nail polish. All of the images were captured with the Olympus VS120 virtual microscopy slide scanning system (Olympus, Tokyo, Japan).

### 4.9. Data Analysis

SPSS (version 13.0) and Origin 9.0 were used for data analysis (student’s *t*-tests) and statistical graphs, respectively. All data were presented as means ± SEM. Statistical significance was set as *, *p* < 0.05, **, *p* < 0.01 and ***, *p* < 0.001.

## Figures and Tables

**Table 1:** The HSV1 genes edited by the CRISPR/Cas9 of the CLEAR strategy and the designed sgRNA sequences.

Target Gene	ORF Position	sgRNA Name	Targeting Sites	sgRNA Sequence (PAM Site)
*VP16 (UL48)*	103,545–105,017, 1473 bp, 65% GC	αVP16-gRNA1	104998–105017	tcgtcgaccaagaggtccattgg
		αVP16-gRNA2	104898–104917	cccctccgctgtacgcaacgggg
*ICP27 (UL54)*	113674–115212, 1539 bp, 69% GC	αICP27-gRNA1	113796–113815	ggagtgttcctcgtcggacgagg
		αICP27-gRNA2	113692–113710	atgctaattgacctcggcctgg
*ICP4 (RS1)*	127082–130990, 146952–150860, 3909 bp, 65% GC	αICP4-gRNA1	C130984–131003	ccccgcatcggcgatggcgtcgg
			C146939–146958	
		αICP4-gRNA2	C130814–130833	ccgggcgtcgtcgaggtcgtggg
			C147109–147128	
*gD (US6)*	138303–139487, 1185 bp, 65% GC	αgD-gRNA1	138368–138387	ggtccgcggcaaatatgccttgg
		αgD-gRNA2	138411–138430	gccgaccccaatcgctttcgcgg
		αgD-gRNA3	139069–139088	cttcaagctgtatacggcgacgg
		αgD-gRNA4	139118–139137	gcagcagggtgctcgtgtatggg

## Data Availability

All data generated or analyzed during this study are included in this published article and its Supplementary Information Files.

## Supplementary Materials

The following supporting information can be downloaded at: https://www.mdpi.com/article/10.3390/pathogens12060814/s1, Figure S1: The inhibitory effect of CRISPR editing on HSV replication in HEK-293T cell line. Figure S2: The control mice received either PBS or LV-Vec showed strong H129-EGFP replication and spread. Figure S3: The effect of suppressing HSV replication in mice subjected to single LV treatments. Figure S4: The effect of suppressing HSV replication in mice from various LV-Cocktail treatment groups.
