# Supplementary material for: CLEAR Strategy Inhibited HSV Proliferation Using Viral Vectors Delivered CRISPR-Cas9
Source: Pathogens. 2023 Jun 7;12(6):814. doi: 10.3390/pathogens12060814 (PMC10302303; doi:10.3390/pathogens12060814)
Supplement: Supplementary file 1 [file pathogens-12-00814-s001.zip › pathogens-2376495 Supplementary Information.pdf]

CLEAR Strategy Inhibited HSV Proliferation Using Viral Vectors Delivered CRISPR-Cas9

Min Ying [1,2,3,4,†], Huadong Wang [2,3,4,*,†], Tongtan Liu [2,3], Zengpeng Han [1,2,3,4], Kunzhang Lin [2,3], Qing Shi [2,3,5], Ning Zheng [1], Tao Ye [6], Huinan Gong [2,3,7] and Fuqiang Xu [1,2,3,4,8,*]

* Correspondence: hd.wang@siat.ac.cn (H.W.), fq.xu@siat.ac.cn (F.X.)
† These authors contributed equally to this work.

Supplementary Information

(Contains Supplementary Figure S1, S2, S3 and S4 with Legend)

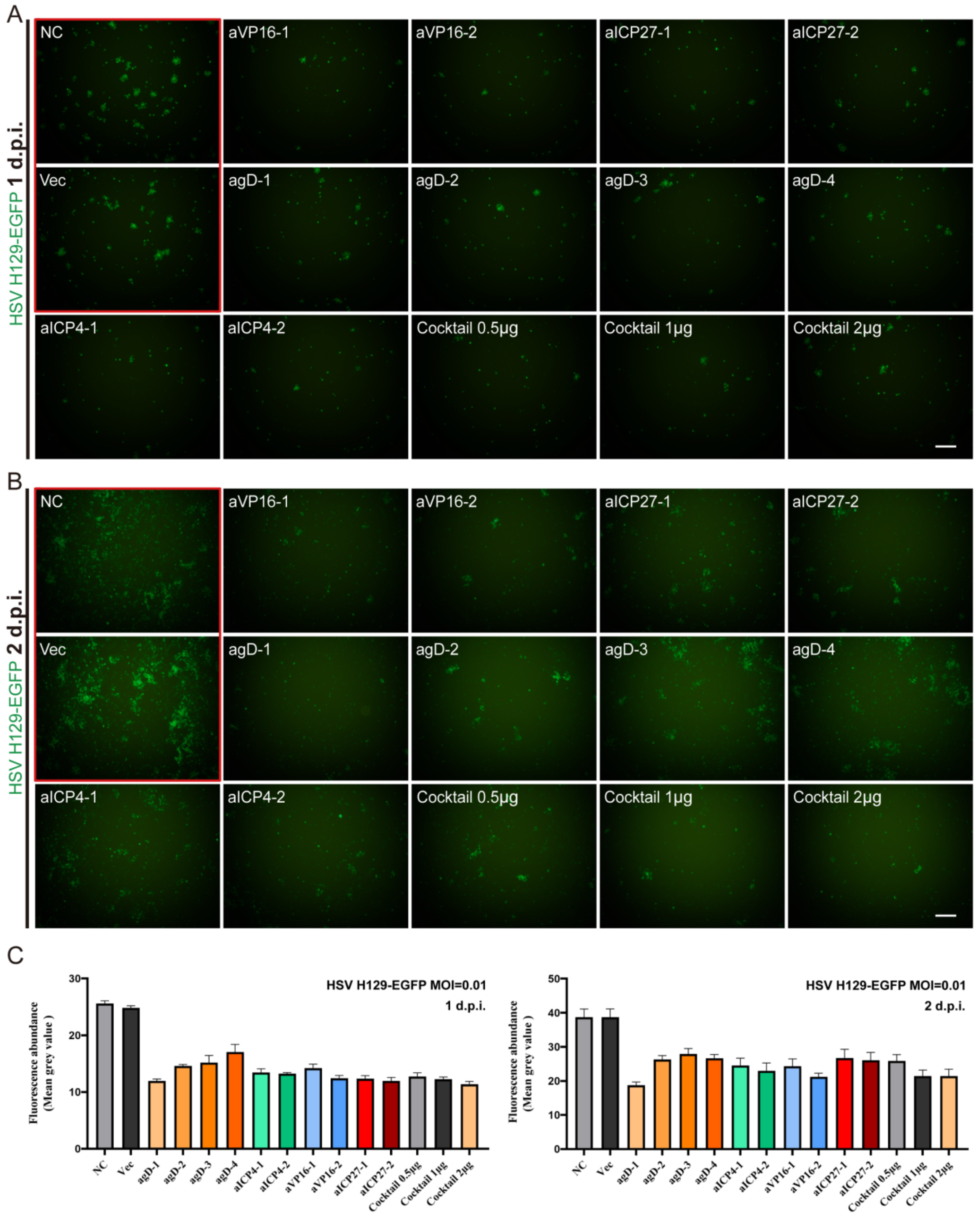

Figure S1. The inhibitory effect of CRISPR editing on HSV replication in HEK-293T cell line. (**A**) The cell fluorescence images of 24h after HSV (H129-EGFP) infection (MOI=0.01), Scale bar = 200 μm; (**B**) The cell fluorescence images of 48h after HSV (H129-EGFP) infection (MOI=0.01), Scale bar = 200 μm; (**C**) The fluorescence abundance (average gray value) of EGFP at 24 and 48h post-infection. The inhibition effect of CRISPR editing on HSV replication was tested in 293T cell line. Different Cas9/gRNA plasmids were transfected into 293T cells, and 24 hours later, cells were infected with HSV H129-EGFP at a multiplicity of infection (MOI) of 0.01 just as same as the experiment on BHK-21 cells. It was found that agD-1, aICP4-2, aVP16-2 and aICP27-2 gRNA plasmids had lower fluorescence abundance by counting the fluorescence abundance of EGFP expression on the first and second day after infection. The results showed that the inhibitory effect of Cas9-gRNAs editing in HEK-293T cells was consistent with the effect in BHK-21 cells.

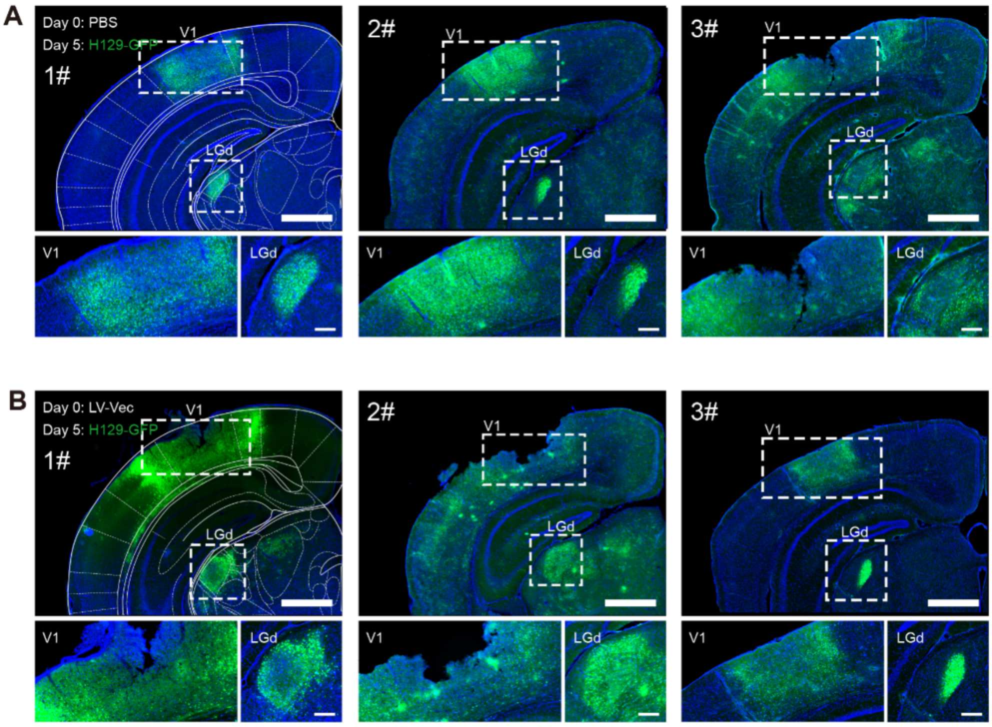

Figure S2. The control mice received either PBS or LV-Vec showed strong H129-EGFP replication and spread. (**A**) HSV1 H129-EGFP viral spread in the PBS group. PBS (500nL) and H129-EGFP ($5×10^2$ PFU) were injected 5 days apart (n = 3). Each panel represents an individual mouse, demonstrating the consistency of HSV1 H129-EGFP inoculation efficiency. Large scale bar = 1 mm, small scale bar = 200 µm. (**B**) HSV1 H129-EGFP viral spread in the LV-Vec group. LV vector control (LV-Vec, $5×10^4$ TU) and H129-EGFP ($5×10^2$ PFU) were injected 5 days apart (n = 3). Each panel displays the EGFP fluorescence in the brain of an individual mouse treated with LV-Vec, illustrating the consistency of HSV1 H129-EGFP inoculation efficiency. Large scale bar = 1 mm, small scale bar = 200 µm.

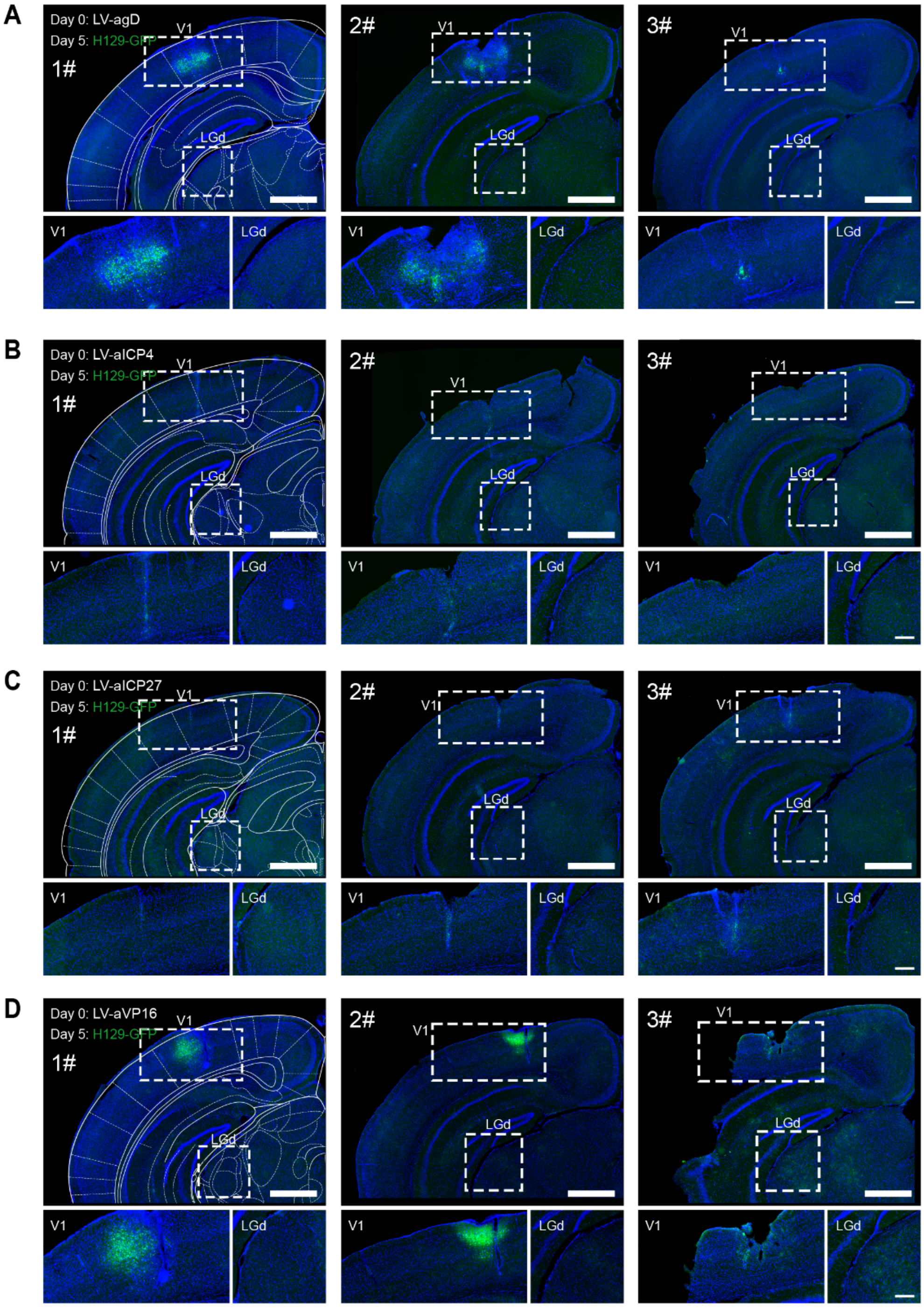

Figure S3. The effect of suppressing HSV replication in mice subjected to single LV treatments. Each Single LV (5×10^4 TU) and H129-EGFP (5×10^2 PFU) were injected 5 days apart (n = 3). (**A**) HSV1 H129-EGFP viral spread in the LV-agD group. (**B**) HSV1 H129-EGFP viral spread in the LV-aICP4 group. (**C**) HSV1 H129-EGFP viral spread in the LV-aICP27 group. (**D**) HSV1 H129-EGFP viral spread in the LV-aVP16 group. Each panel represents an individual mouse, displaying the distribution of EGFP fluorescence at the injection site of V1 and downstream areas of LGd via the single administrated LV. Large scale bar = 1 mm, small scale bar = 200 μm.

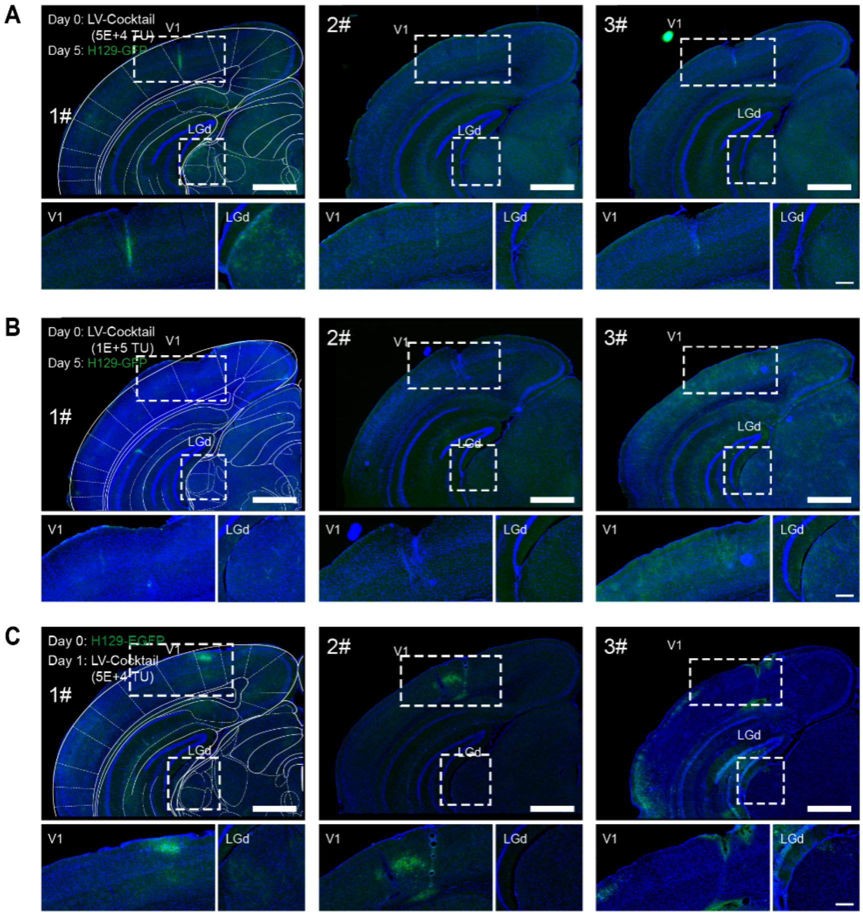

Figure S4. The effect of suppressing HSV replication in mice from various LV-Cocktail treatment groups. (**A**) HSV1 H129-EGFP viral spread in the group treated with LV-Cocktail (5×10[4] TU) first, followed by H129-EGFP (5×10[2] PFU) administration 5 days apart (n = 3). (**B**) HSV1 H129-EGFP viral spread in the double-concentration mixed treatment group that received LV-Cocktail (1×10[5] TU) first, followed by H129-EGFP (5×10[2] PFU) administration 5 days apart (n = 3). (**C**) HSV1 H129-EGFP viral spread in the group inoculated with H129-EGFP (5×10[2] PFU) first, followed by LV-Cocktail (5×10[4] TU) administration 1 day later (n = 3). Each panel represents an individual mouse, displaying the distribution of EGFP fluorescence at the injection site of V1 and downstream areas of LGd via the LV-Cocktail group. Large scale bar = 1 mm, small scale bar = 200 μm.
